# Cardiac arrhythmia in individuals with steroid sulfatase deficiency (X-linked ichthyosis): candidate anatomical and biochemical pathways

**DOI:** 10.1042/EBC20230098

**Published:** 2024-12-04

**Authors:** Georgina H. Wren, William Davies

**Affiliations:** 1 School of Psychology, Cardiff University, Cardiff, U.K.; 2 Division of Psychological Medicine and Clinical Neurosciences and Centre for Neuropsychiatric Genetics and Genomics, School of Medicine, Cardiff University, Cardiff, U.K.; 3 Neuroscience and Mental Health Innovation Institute, Cardiff University, Cardiff, U.K.

**Keywords:** Cellular communication network (CCN) factor, dehydroepiandrosterone sulfate (DHEAS), laminin, sex hormones, ventricular

## Abstract

Circulating steroids, including sex hormones, can affect cardiac development and function. In mammals, steroid sulfatase (STS) is the enzyme solely responsible for cleaving sulfate groups from various steroid molecules, thereby altering their activity and water solubility. Recent studies have indicated that Xp22.31 genetic deletions encompassing *STS* (associated with the rare dermatological condition X-linked ichthyosis), and common variants within the *STS* gene, are associated with a markedly elevated risk of cardiac arrhythmias, notably atrial fibrillation/flutter. Here, we consider emerging basic science and clinical findings which implicate structural heart abnormalities (notably septal defects) as a mediator of this heightened risk, and propose candidate cellular and biochemical mechanisms. Finally, we consider how the biological link between STS activity and heart structure/function might be investigated further and the clinical implications of work in this area.

## Sex hormones and the cardiovascular system

Sex hormones including androgens, oestrogens and progestogens, are synthesized from cholesterol through the process of steroidogenesis [1]. Such hormones can have a variety of diverse array of effects on the developing and mature cardiovascular system including on cell proliferation and death, contractility, fibrosis and reactive oxygen species generation [1]. Additionally, given that they differ in levels across the sexes throughout development, sex hormones are likely to contribute to sex differences in cardiovascular phenotypes, including with respect to disease risk [2,3]. Recent studies have shown that estrogen-related receptor and progesterone receptor-mediated signaling cascades are particularly important in cardiac maturation processes, and the transition from the fetal to the postnatal and adult heart [4,5].

## A brief introduction to steroid sulfatase

In mammals, the steroid sulfatase (STS) enzyme is solely responsible for cleaving sulfate groups from conjugated steroids, including: cholesterol, progesterone, dehydroepiandrosterone (DHEA) and estrone sulfates [6]. The desulfation process, typically occurring in target tissues, acts to alter the activity of the compounds, and to lower their water solubility and stability [7]. Many of the so-called ‘free’ steroids generated can subsequently act as precursors for a variety of oestrogens and androgens [7]. *STS* is expressed at highest levels in the adult arterial vasculature and in the placenta, and at comparatively lower levels in multiple other tissues during development and postnatally, including in the brain, heart, and gastrointestinal tract [7–9].

## Clinical consequences of STS deficiency

In humans, the gene encoding STS is located on the short arm of the X chromosome at Xp22.31. Genetic deletions encompassing *STS* (typically approximately 1.6 Mb in size) are thought to be present in around 1 in 1500 males and 1 in 750 females [10]. Such deletions, or nonsense/mis-sense single nucleotide variants within *STS*, are associated with the rare dermatological condition X-linked ichthyosis (XLI) [11]. XLI is characterized by skin scaling as a consequence of abnormal desquamation and retention hyperkeratosis due to excessive cholesterol sulfate accumulation in the stratum corneum [12]. Xp22.31 deletions including *STS* also appear to be associated with an increased likelihood of cryptorchidism, benign corneal opacities, and neurodevelopmental, hemostatic, and fibrotic conditions [13,14]. Using the large UK Biobank (UKBB) sample, it has recently been shown that middle-aged males with genetic deletions at Xp22.31 are around four times more likely to have been diagnosed with atrial fibrillation/flutter (AF) than similarly-aged non-carrier males (approximately 10% of deletion carriers vs. 2.5% of non-carriers); however, diagnoses of AF occurred at similar frequencies in deletion and non-deletion carrying females [15]. In terms of pathology, of the deletion-carrying males with AF within the UKBB sample, one had a relevant cardiovascular ICD-10 descriptor (‘left ventricular failure’) and all three deletion-carrying females with AF had been diagnosed with ‘left ventricular failure’ [16].

Atrial fibrillation is the most common form of supraventricular tachycardia, and is characterized by irregular and rapid beating of the atria; atrial flutter is also characterized by an elevated atrial contraction rate, although this tends to be more regular than for atrial fibrillation [17]. Both atrial fibrillation and flutter can lead to turbulent blood flow through the heart, increasing the likelihood of thombosis and embolism, together with downstream cardiac and neurological complications such as heart failure, stroke and accelerated cognitive decline/dementia [17]. Risk of AF is potentiated by the existence, or co-occurrence, of a number of conditions including: (congenital) structural heart defects, hypertension, hypercholesterolemia/coronary artery disease, cardiac or systemic infection/inflammation, anemia, metabolic disorders (notably diabetes), and obesity [17]. Taking into account the prevalence of Xp22.31 deletions within the general population, and associated effect sizes, it has been estimated that this Copy Number Variant might explain around 1 in 300 cases of idiopathic AF in middle-aged males, equivalent to around 650 new UK cases per year, and around 10,000 new US cases annually [17]. Reliably identifying individuals at risk of AF and revealing associated pathology at an early stage will be key to ensuring optimal intervention and the best possible clinical outcomes for patients.

Work building upon the UKBB study has indicated that self-reported abnormal heart rhythms are common in both males with XLI and female carriers of XLI-associated genetic variants (>25% of cases); these abnormal rhythms tended to be precipitated by stress, and generally resolved within 24 h [16]. In males with XLI, ‘gut (gastrointestinal) issues’ were reported more commonly in individuals with heart rhythm abnormalities than those without, whilst amongst female carriers, ’heart murmur’, ‘anemia,’ and ‘asthma’ were reported more frequently in individuals reporting heart rhythm abnormalities than those not reporting these [16]. Association analyses in UKBB across the Xp22.31 consensus deletion interval in cases of idiopathic AF and unaffected individuals identified an excess of common genetic risk variants in *STS* only, implicating this specific gene in the AF phenotype [16].

Theoretically, STS (and its deficiency) could influence cardiac (dys)function through direct effects on heart tissue, or through indirect effects via the endocrine system. The link between STS deficiency and AF risk could be mediated by a combination of one or more of the following mechanisms: (i) an imbalance between levels of sulfated and free steroids e.g. DHEA(S) or cholesterol (sulfate), (ii) induction of pro-fibrotic factors such as Cellular Communication Network (CCN) proteins, (iii) effects on cholinergic neurotransmission and iv) vulnerability to valvular stenosis [17]. Exciting new data have suggested a novel anatomical risk mechanism consistent with these previously proposed risk pathways.

## A new risk mechanism?

McGeoghan and colleagues investigated the effects of knocking down *STS* expression in human primary differentiated keratinocyte cell cultures on gene expression, and described the biological pathways affected by the manipulation [18]. Genes that were upregulated by the experimental manipulation clustered into a number of interesting pathways, including ‘regulation of blood coagulation/haemostasis,’ providing converging evidence for STS deficiency predisposing to haemostatic abnormalities. Genes that were down-regulated clustered within pathways including ‘astrocyte development’ (of potential relevance to neurodevelopmental condition vulnerability in XLI) and ‘keratinocyte differentiation/keratinization’ (of possible relevance to the condition’s desquamating/retention hyperkeratosis phenotype). Of particular interest with relevance to cardiac biology was the finding that amongst the genes downregulated upon *STS* knockdown, there was significant enrichment for those involved in ‘cardiac/ventricular septum morphogenesis.’ This interesting finding (albeit in skin, rather than cardiac, cells) suggests the possibility that STS deficiency may somehow confer vulnerability to cardiac septal defects and thereafter to arrhythmias.

Congenital septal defects arise as a consequence of aberrant early embryogenic processes [19]. Combined, ventricular and atrial septal defects have been observed in around 50/10,000 (0.5%) live or stillbirths from the general USA population surviving until at least 20 weeks' gestation [20]. They can lead to impaired cardiac function and ultimately heart failure through ventricular volume overload and resultant hypertrophy; the left ventricle is particularly, but not exclusively, affected [21–23]. Smaller defects (<5 mm) may be asymptomatic and can repair spontaneously during development. However, larger defects are associated with symptoms including cyanosis, breathlessness, tachycardia and fatigue as a consequence of blood bypassing the lungs and becoming increasingly deoxygenated, and, as such, often require surgical intervention [21,22]. Septal defects can be detected and characterised through a combination of cardiac auscultation and echocardiography; classically, ventricular septal defects cause a holosystolic or pansystolic murmur [21,22]. Ventricular septal defects are most often perimembranous, occurring close to the valves, and can co-occur with atrial septal defects [21,22]. There is a well-established link between septal defects and atrial fibrillation occurring later in life, even when the defects have been repaired [24–27]. A key question is whether there is any existing evidence for a significant association between septal defects and Xp22.31 deficiency.

## Cardiac septal defects associated with Xp22.31 deletions or X-linked ichthyosis

The DECIPHER database is a repository containing information on a large number of cases ascertained by an international network of academic clinical genetics departments, including our departments at University Hospital of Wales and Neuroscience and Mental Health Innovation Institute at Cardiff University [28]. Of 68, predominantly young, male individuals in this database with Xp22.31 deletions of <10 Mb encompassing *STS*, for which phenotypic descriptors are freely-available, three (4.4%) are reported as presenting with cardiac abnormalities: one with ‘abnormal mitral valve morphology’ and ‘perimembranous ventricular septal defect,’ one with ‘abnormal heart morphology’ (not further specified), and one with ‘ventricular septal defect.’ Of 41 deletion-carrying female heterozygotes meeting the same criteria, only one (2.4%) has documented cardiac issues: ‘cardiomyopathy’ and ‘congenital malformation of the right heart’ (not further specified). The four individuals with reported cardiac abnormalities possessed deletions of the size similar to, or smaller than, those typically associated with XLI (1.32–1.64 Mb).

In addition, there are a number of cases with Xp22.31 deletions and/or X-linked ichthyosis who have been described in the literature. Baspinar and colleagues describe a two-month-old boy with X-linked ichthyosis presenting with mirror-image dextrocardia, a large perimembranous ventricular septal defect, and sub-valvular aortic stenosis upon transthoracic echocardiography; he also presented with moderate pulmonary hypertension [29]. His twin sister did not exhibit skin issues, nor any evidence of cardiac disease. Xu et al. described two individuals within a monozygotic female twin pair, both of whom were heterozygous for a 1.6 Mb deletion, one of whom presented with a ventricular septal defect [30]. Hu et al. reported that two of seven liveborn female Xp22.31 deletion carriers identified during prenatal screening exhibited cardiac abnormalities *in utero* (one ventricular septal defect, one echogenic intracardiac focus) but were asymptomatic postnatally [31]; cardiac anomalies were not reported in eight liveborn deletion-carrying males in the same study. Finally, in a conceptually different approach, Meerschaut and co-workers analyzed Copy Number Variants in 270 children (158 boys and 112 girls) diagnosed with congenital heart defects; they identified one female Xp22.31 deletion carrier presenting with such a defect, although its nature was not further specified [32].

In the UKBB, an older participant sample, no deletion carriers were reported as presenting with septal defects (as indexed by ICD-10 descriptors). Potentially, small defects in childhood arising as a consequence of Xp22.31 deletion may have spontaneously closed and may be undetectable/unremarkable in middle-age. Our ongoing studies with male participants affected by XLI, and female carriers, have identified several adults with a history of atrial or ventricular septal defects, some of whom have presented with an irregular heartbeat (notably premature ventricular contractions (PVCs). Unusually high numbers of PVCs have previously been described in >50% of middle-aged individuals with repaired or unrepaired ventricular septal defects [33].

Despite limited data, the rate of presentation of septal defects in Xp22.31 deletion carriers appears somewhat higher than might be anticipated on the basis of prevalence rates for idiopathic defects; however, it is important to recognize that this excess might be influenced to some extent by publication and reporting biases.

## Candidate hormonal, cellular and biochemical pathways

If STS deficiency is genuinely associated with an increased likelihood of cardiac structural defects, what might the mediating biological mechanisms be? The proximal effects of loss of enzyme function are likely to be on levels of sulfated and free steroid hormones, and the balance between these. DHEAS is the most abundant circulating steroid in humans [34], and there is some evidence, albeit fairly weak and correlational, that DHEAS levels are associated with aspects of cardiac structure. Szathmari and colleagues reported a positive correlation between DHEAS serum levels and left ventricular mass index (LVMI) in pre-menopausal women being treated for essential hypertension, and an inverse correlation between DHEAS serum levels and both LVMI and ventricular septal thickness in post-menopausal women with essential hypertension [35]; the authors speculated that their findings might reflect DHEA(S)-dependent effects on fibroblast proliferation [36–38]. More recently, Cedars et al. demonstrated that, in adults with congenital heart disease (ACHD), lower levels of DHEA were associated with right ventricular (RV) dysfunction and hypothesised that ‘breakdown of a homeostatic signaling mechanism involving DHEA may contribute to propagation of RV dysfunction in ACHD’ [39].

**Figure 1:**
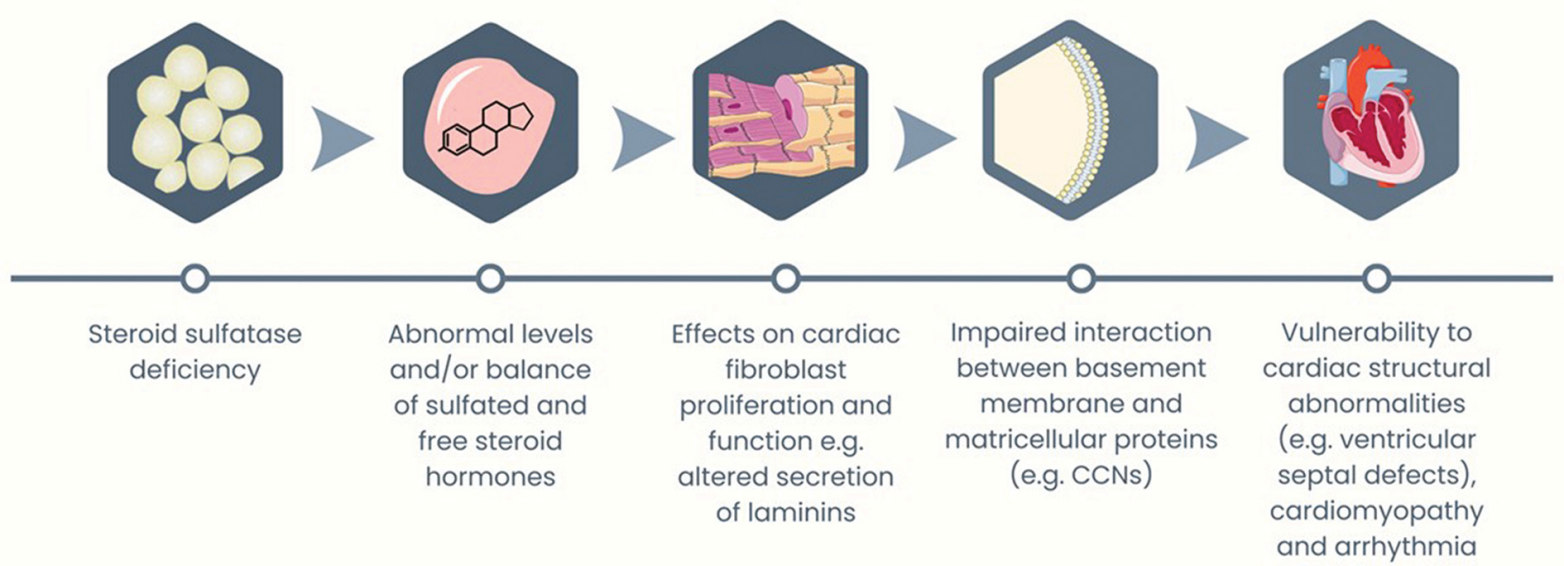
A theoretical model linking steroid sulfatase deficiency to increased vulnerability to cardiac arrhythmias Icons by Servier https://smart.servier.com/ is licensed under CC-BY 3.0 Unported https://creativecommons.org/licenses/by/3.0/

We have previously argued that the cutaneous and extracutaneous phenotypes associated with XLI may be partially explained by aberrant basement membrane function, and impaired interaction between the basement membrane and matricellular proteins [14]; perturbed interactions between basement membrane-associated laminin proteins and matricellular Cellular Communication Network (CCN) factor proteins (the expression of which is sensitive to acute STS inhibition) may be particularly significant [14,40,41]. Cardiac fibroblasts secrete laminins [42], and intact basement membrane/laminin function is key to healthy cardiac and septal development [43]. We thus propose a model whereby loss of STS, via effects on systemic steroid sulfate levels, disrupts cardiac fibroblast proliferation, laminin secretion and basement membrane-CCN protein interactions; these endocrinological and cellular processes then culminate in vulnerability to septal defects, ventricular hypertrophy, and increased arrhythmia risk (Figure 1). In support of this model: (i) there is significant down-regulation of fibroblast and septal-morphogenesis-related genes, notably Fibroblast Growth Factor Receptor 2 (*FGFR2*), in skin cells with compromised STS activity [18], (ii) *LAMC1* genetic variants cause Dandy-Walker syndrome (frequently linked to atrial or ventricular septal defects) [44,45], (iii) deletion of the *Ccn3* gene in mice results in enlargement and abnormal modeling of the endocardial cushions, septal defects, and ventricular hypertrophic cardiomyopathy [46], and (iv) deletion of *Ccn1* in mice results in impaired cardiac valvuloseptal morphogenesis [47].

## Future work and conclusions

Further work is required in human participants to investigate the relationships between Xp22.31 deletions (or *STS*-specific single nucleotide variants), steroid hormone levels, and cardiac morphology/function. In particular, systematic collection of clinical information on cardiac investigations and findings (auscultation, echocardiography, and electrocardiography) in individuals presenting with XLI and arrhythmias would be helpful. Interrogating large biobanks where relevant genetic, biomarker, and cardiac structural/functional measurements are routinely collected should enable these analyses and provide maximal experimental power; to date, interventricular septum morphology and baseline ECG readings have been taken in 32–40,000 UKBB participants [48,49]. Such analyses in man may be complemented by work in experimentally tractable model organisms where the molecular and cellular disruptions arising from STS deficiency, and the developmental consequences of these, may be probed. For many years, the genetic architecture around the *Sts* locus in mice precluded the generation of gene-specific knockout mice by traditional methods; new approaches such as CRISPR have enabled the development of mice with *Sts*-specific null mutations. A recent study in an *Sts*-deletion mouse has implicated dysregulated Wnt/β and Hippo signaling, and excessive reactive oxygen species production, in the abnormal skin phenotype observed in this model [50]; disruption to these pathways has previously been suggested to contribute towards cardiac disease risk [51,52] and they warrant further examination in individuals with XLI, in rodent models and in non-mammalian models such as zebrafish [53,54]. Together, these cross-species studies will clarify the cardiac sequelae of STS deficiency, and may provide clues as to how best to identify and manage any abnormalities. Given that the *STS* gene (and associated enzyme) is expressed more highly in female than male tissues as a consequence of its escape from X-inactivation, these studies might also shed light upon the sex-biased nature of cardiac arrhythmia risk [17].

## Summary

- Steroid hormones, including sex hormones, can influence cardiac development and function. The steroid sulfatase (STS) enzyme cleaves sulfate groups from a variety of steroid hormones.
- Loss of STS function is associated with a considerably increased risk of cardiac arrhythmia.
- Recent data from cellular models in which STS is knocked down, and from clinical cases lacking STS constitutively, have implicated abnormal septal development as a potential mediator of arrhythmia risk.
- Cardiac phenotypes in STS-deficient individuals may be due to perturbed basement membrane function and abnormal interactions with matricellular proteins.
- Investigation of cardiac phenotypes (notably septal morphology) in STS-deficient individuals, and in new STS-deficient animal and cellular models, should clarify the genotype-phenotype relationship and provide clinically-useful information to cardiologists.

## Open Access

Open access for this article was enabled by the participation of Cardiff University in an all-inclusive *Read & Publish* agreement with Portland Press and the Biochemical Society under a transformative agreement with JISC.

## Abbreviations

ACHD: adults with congenital heart disease
AF: atrial fibrillation/flutter
CCN: Cellular Communication Network
DHEA: dehydroepiandrosterone
LVMI: left ventricular mass index
PVC: premature ventricular contraction
RV: right ventricular
STS: steroid sulfatase
